# Safety, Tolerability, and Serum/Tear Pharmacokinetics of Human Recombinant Epidermal Growth Factor Eyedrops in Healthy Subjects

**DOI:** 10.3390/ph15111312

**Published:** 2022-10-24

**Authors:** Hyounggyoon Yoo, Seonghae Yoon, In-Jin Jang, Kyung-Sang Yu, Joon Young Hyon, Jungi Hwang, Inyoung Hwang, Jung Sunwoo, Jae-Yong Chung

**Affiliations:** 1 Department of Clinical Pharmacology and Therapeutics, Seoul National University College of Medicine, Seoul 03080, Korea; 2 Department of Clinical Pharmacology and Therapeutics, CHA Bundang Medical Center, Seongnam 13520, Korea; 3 Department of Clinical Pharmacology and Therapeutics, Seoul National University Bundang Hospital, Seongnam 13620, Korea; 4 Department of Clinical Pharmacology and Therapeutics, Seoul National University Hospital, Seoul 03080, Korea; 5 Department of Ophthalmology, Seoul National University College of Medicine, Seoul 03080, Korea; 6 Department of Ophthalmology, Seoul National University Bundang Hospital, Seongnam 13620, Korea; 7 Department of Clinical Pharmacology and Therapeutics, Chungbuk National University Hospital, Cheongju 28644, Korea; 8 Clinical Trials Center, Chungnam National University Hospital, Daejeon 35015, Korea

**Keywords:** dry eye disease, epidermal growth factor, first-in-human study, pharmacokinetics

## Abstract

The purpose of this study was to evaluate the safety, tolerability, and pharmacokinetics (PKs) of rhEGF eyedrops after the administration of single and multiple doses in healthy subjects. A phase 1, randomized, double-blind, placebo-controlled, and single-ascending dose (SAD) and multiple-ascending dose (MAD) study were conducted in three dose groups (10, 50, and 100 μg/mL). The subjects randomly received rhEGF eyedrops or the placebo in a 3:1 ratio. Serial blood and tear samples for PK analysis were collected up to 36 h and 180 h post-dose in SAD and MAD studies, respectively. In addition, the serum and tear EGF concentrations were measured. Immunogenicity evaluations were conducted using serum anti-EGF antibody levels. A total of 50 subjects were enrolled and 48 subjects completed the study. Adverse drug reactions were mild and transient. There were no serious adverse events in this study. The tear EGF concentrations rapidly increased and returned to baseline after 4 h without any serum EGF level change after the administration of rhEGF eyedrops. rhEGF eyedrops were safe and well-tolerated in healthy subjects in a dose range of 10–100 μg/mL, indicating suitability for further studies in patients with corneal injury.

## 1. Introduction

Corneal injury is a common disease, accounting for approximately 3% of emergency department visits [1]. The cornea can be injured by numerous causes including oculopathy, mechanical trauma, infection, inflammation, chemicals, and radiation [2]. Corneal injuries are important because they may be vision-threatening. However, there are no absolute medical therapies for it. 

Corneal healing is a complex process involving cell death, migration, proliferation, differentiation, and extracellular matrix remodeling [3]. In addition, limbal stem cells and basement membrane remodeling play key roles in corneal healing [4]. Several cytokines, chemokines, and growth factors are involved in corneal healing. 

Epidermal growth factor (EGF), one of the growth factors that are secreted from the lacrimal gland [5], is involved in corneal healing; it regenerates limbal stem cells and regulates the migration of corneal cells, thus, accelerating corneal healing [6,7]. In addition, previous studies showed that tear EGF concentration was significantly decreased in dry eye syndrome, which is the one of most common causes of corneal injury [8]. Therefore, EGF has been thought to be a treatment option for corneal injury.

For the treatment of corneal injury, the topical eye administration of EGF has been used through cord blood serum, autologous serum, amniotic membrane extract, and amniotic membrane transplantation [9,10,11,12]. However, the currently implemented treatment options may not be widely used because the production of these products as treatment options is difficult, and the infection risk is relatively high. Therefore, human recombinant EGF (rhEGF) eyedrops could be an appropriate treatment option to solve the above problems.

Some studies showed the effect of rhEGF eyedrops in vitro and animal studies [7,13]. However, there are no published human data about rhEGF eyedrops. The purpose of this study was to evaluate the safety and tolerability and pharmacokinetics (PKs) of EGF in serum and tears after topical administration of single and multiple doses of rhEGF eyedrops in healthy subjects.

## 2. Results

### 2.1. Demography

Fifty subjects (twenty-five each in the single ascending dose (SAD) and the multiple ascending dose (MAD) study) were enrolled. A total of 48 subjects completed this study as planned. Two subjects (one each in SAD and MAD study) dropped out due to the withdrawal of their own consent. There were no significant differences in demographic characteristics among the dose groups in SAD and MAD studies, respectively (Supplementary Table S1).

### 2.2. Safety

rhEGF eyedrops were well tolerated in all dose groups in SAD and MAD studies. There were six adverse drug reactions (ADRs) by five subjects treated with rhEGF eyedrops and two ADRs by two subjects treated with a placebo in SAD study, and six ADRs by five subjects treated with rhEGF eyedrops and five ADRs by four subjects with a placebo in MAD study, respectively (Table 1). All ADRs were mild and transient, and there were no serious adverse events. The most common ADR was corneal erosion, which probably resulted from the tear sampling procedure, which was reported six times by six subjects and eight times by seven subjects in the SAD and MAD studies, respectively. There were no significant differences in the incidences of ADRs in SAD (*p*-value 0.7660. Fisher’s exact test) and MAD (*p*-value 0.4631. Fisher’s exact test) studies, respectively. 

There were no clinically significant findings for vital signs, physical examinations, and ECG. In addition, there were no changes in ophthalmic examinations after the administration of rhEGF eyedrops (Supplementary Table S2).

### 2.3. Pharmacokinetics

**Figure 1:**
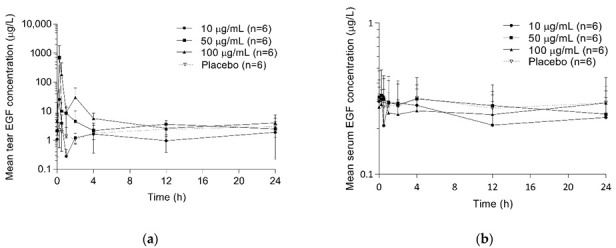
Mean EGF concentration-time curves after single administration of rhEGF eyedrops: (**a**) tear; (**b**) serum.

**Figure 2:**
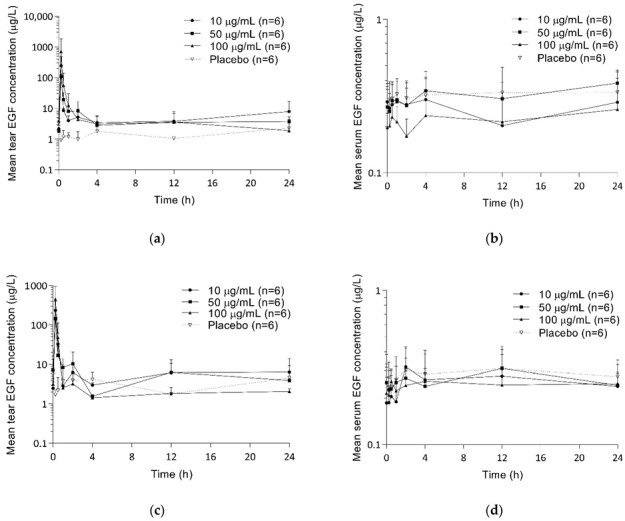
Mean EGF concentration-time curves after multiple administration of EGF eyedrops: (**a**) tear, day 1; (**b**) serum, day 1; (**c**) tear, day 14; (**d**) serum, day 14.

After the administration of rhEGF eyedrops, the mean serum EGF concentration showed numerous peaks, but it did not change compared to those of baseline and placebo, while the mean tear EGF concentrations increased compared to those of baseline and placebo in SAD and MAD studies (Figure 1 and Figure 2). rhEGF eyedrops were rapidly absorbed with the time to reach the maximum concentrations (T_max_) of tear at ranges of 0.18–0.2 and 0.18–0.27 h in SAD and MAD studies, respectively (Table 2 and Table 3). Then, the mean tear EGF concentrations became similar compared to those of baseline and placebo after 4 h of administration in SAD and MAD studies. The maximum concentrations (C_max_) of tear and the area under the concentration-time curve from the pre-dose to 12 h (AUC_0-12h_) increased by dose in SAD and MAD studies. However, The observed serum concentration after 12 h of the administration (C_12h_) of tear was not changed by rhEGF eyedrops in SAD and MAD studies.

### 2.4. Immunogenicity

After the administration of rhEGF eyedrops, no anti-EGF antibodies were detected in SAD and MAD studies. Although anti-EGF antibodies were detected in a subject in the placebo group before the administration of rhEGF eyedrops, they were not detected after the administration of the placebo. (Table S3).

## 3. Discussion

This study was the first-in-human study to evaluate the safety/tolerability and PK of rhEGF eyedrops after single and multiple administrations in healthy male subjects. There were some published EGF eyedrops in corneal injury including dry eye syndrome; however, there have been no PK studies of EGF eyedrops [14]. Therefore, this study was the first published PK study for rhEGF eyedrops.

One of the primary goals of first-in-human study was to determine safety/tolerability. Considering the safety profiles, rhEGF eyedrops at doses ranging from 10 μg/mL to 100 μg/mL in SAD and MAD studies were well tolerated. No subjects experienced serious adverse events. Among subjects who received rhEGF, corneal erosion was the most common ADR. The incidences of corneal erosion were similar among dose groups, including placebo. In addition, corneal erosions occurred on the medioinferior side, in which tear sampling was commonly conducted. Therefore, corneal erosion may be caused by tear sampling procedures rather than rhEGF eyedrops. 

After administrations of rhEGF eyedrops, there were numerous peaks in time-serum EGF concentration profiles. Serum EGF levels show high variability through numerous causes, including circadian rhythm, ultradian rhythm, and venous puncture [15,16]. In addition, normal serum EGF levels are 0.1–1.281 μg/L [16,17]. In this study, all of the serum EGF levels were within the normal serum EGF levels. Therefore, the numerous peaks are thought to be due to ultradian rhythm rather than rhEGF eyedrops, and rhEGF eyedrops did not affect serum EGF levels. Given that elevated blood EGF levels are linked with several toxicities, such as esophageal adenocarcinoma and non-small lung cancer, rhEGF eyedrops may be delivered without causing systemic toxicity [17,18,19,20].

In this study, tear EGF was rapidly absorbed; then, tear EGF concentrations became similar compared to those of baseline and placebo after 4 h without accumulation. The rapid decline of tear EGF concentrations may result from precorneal fluid drainage, including nasolacrimal drainage and blinking despite the lack of clear mechanisms of rhEGF binding to ocular tissue [21]. Further study may be needed to find the mechanism of corneal binding of rhEGF after administration of rhEGF eyedrops. The absence of tear EGF accumulation means that the possibility of toxicity due to the accumulation of rhEGF eyedrops is low during long-term administration. 

There has been no published data on therapeutic tear EGF concentrations for corneal injury in humans. Some in vitro studies reported that the therapeutic EGF concentrations were 1–10 μg/L [22,23]. Here, the average tear EGF concentrations among dose groups were 0.74–18.8 μg/L and 2.6–12.4 μg/L in SAD and MAD studies, respectively. Therefore, 50 μg/mL of rhEGF eyedrop twice a day may be considered a therapeutic dose because the average tear EGF concentrations in the dose group of 50 μg/mL were 5.8 μg/L and 4.1 μg/L in SAD and MAD studies, respectively. 

Here, tear EGF exposure did not show dose-linearity although tear EGF exposure increased by dose. The amount of tears may affect the tear EGF concentration, which is thought to have influenced the PK variability of EGF after administration of rhEGF eyedrops. Considering the PK variability and lack of accumulation of rhEGF eyedrops, tear EGF concentration is not thought to be a suitable biomarker for the evaluation of the efficacy of rhEGF eyedrops.

Although rhEGF eyedrops have been emerging as a good treatment option for corneal injury, no appropriate dose of rhEGF eyedrops has been known. There are some studies showing that higher doses of EGF eyedrops might be inappropriate for treating corneal injury because of the possibility of auto-inhibition at a high dose of EGF; however, these studies did not show any dose-tear EGF concentration relationships [22,24,25]. Therefore, further studies for corneal injury patients may be needed considering tear PK variability and the safety profile of rhEGF eyedrops.

In this study, no anti-EGF antibodies were detected except for a subject on a placebo before rhEGF administration. Generally, there are numerous soluble proteins which bind with EGF, including human EGF receptor 1, human EGF receptor 2, and arginine esterase [26,27,28]. Therefore, rhEGF eyedrops might be administered without a decrease in efficacy due to anti-EGF antibody formation even if administered for a long time.

There are some limitations in this study. This study was performed on healthy subjects to minimize confounding factors that could influence the study results. Further studies for corneal injury patients are needed to evaluate the efficacy of the drug. In addition, this study was not conducted for female subjects. Generally, the first-in-human studies for healthy subjects were performed for male subjects because the reproductive toxicity of investigational products has not been sufficiently characterized at early development stages. Therefore, this study was conducted for only male subjects, considering the reproductive toxicity of rhEGF eyedrops had not been sufficiently revealed. There were missing values of tear PK sampling due to the difficulty of the sample collection. Nevertheless, the PK profile could be observed in all dose groups.

## 4. Materials and Methods

This study was conducted at the Seoul National University Bundang Hospital (SNUBH). The study protocol was approved by the Institutional Review Board of SNUBH (No. B-1902/522-005). This study was conducted according to the major ethical principles of the Declaration of Helsinki and Good Clinical Practice Guidelines. All subjects provided their written informed consent before any study-related procedures.

### 4.1. Study Design and Subjects

This study was a phase 1, randomized, double-blind, placebo-controlled, SAD and MAD study. The subjects were enrolled and randomly assigned to the rhEGF eyedrops (10, 50, or 100 μg/mL) or placebo in a 3:1 ratio. The subjects received rhEGF or placebo twice a day for one day and 14 days in the SAD and the MAD studies, respectively. The subjects who were assigned to the rhEGF eyedrops received rhEGF eyedrop in one eye, which was randomly assigned, and a placebo in the other eye. Dose escalation was determined based on the evaluation of safety/tolerability data from the previous dose groups. In addition, whether to proceed to the MAD study was determined based on the safety/tolerability results of the SAD study.

In this study, healthy male subjects who had a body weight of 50–100 kg and body mass index of more than 18.0 kg/m^2^ and were 19–50 years of age were eligible for enrollment in the study if no clinically significant abnormalities were observed in medical history, physical and ophthalmic examinations, clinical laboratory tests, vital signs, and 12-lead electrocardiograms (ECG).

### 4.2. Pharmacokinetic Sampling and Bioanalysis

For PK sampling, serial blood and tear samples of EGF were collected at the scheduled times: pre-dose and 0.25 h, 0.5 h, 1 h, 2 h, 4 h, and 12 h at −1 d and 1 d, and pre-dose and 12 h at 2 d after dosing for dose groups of SAD study; pre-dose and 0.25 h, 0.5 h, 1 h, 2 h, 4 h, and 12 h at −1 d, 1 d, and 14 d, pre-dose at 2 d, 8 d, and pre-dose and 12 h at 15 d after dosing for dose groups of MAD study. Tear samples were collected from the marginal tear strip of the lower lid near the medial canthus by using disposable microcapillaries. 

Serum and tear concentrations of EGF were analyzed by a validated enzyme-linked immunoassay (ELISA) using a human EGF Quantikine ELISA kit (R&D systems, Minneapolis, MN, USA). Serum and tear samples were diluted appropriately in the Assay Diluent RD1-6. EGF calibration standards were prepared at the following concentrations: 3.91, 7.81, 15.6, 31.3, 62.5, 125, and 250 pg/mL. 200 μL of diluted sample or standard was added to the plate and incubated for 2 h at room temperature. 

After the incubation, the plates were washed three times with the wash buffer. Next, 200 μL of human EGF conjugate was added to each well, then, incubated for 2 h at room temperature. After the incubation, the plates were washed three times with the wash buffer. After that, 200 μL of the substrate was added to each well and incubated for 20 min at room temperature. Then, 50 μL of reaction termination solution was added to each well to terminate the reaction. The subsequent absorbance was quantified by measurement at 450 nm using a VersaMax Microplate Reader (Molecular Devices, San Jose, CA, USA) and the results were analyzed using Softmax Pro 7.1 GxP (Molecular Devices, San Jose, CA, USA).

### 4.3. Pharmacokinetic Analysis

Noncompartmental analysis was performed to calculate the PK parameters of serum and tear EGF using Phoenix^®^ WinNonlin^®^ software version 8.0 (Certara, St. Louis, MO, USA). The observed serum concentrations and times were used to estimate the C_max_ and C_12h_ of serum and tear EGF and T_max_. AUC_0-12h_ was calculated using the linear up/log down trapezoidal method.

### 4.4. Immunogenicity Evaluations

Anti-EGF antibodies were determined for assessment of immunogenicity from serum samples at the scheduled times; prior to following the rhEGF eyedrops administration at week 4 in the SAD study and prior to following the rhEGF eyedrops administration at weeks 1, 2, and 6 in the MAD study. The anti-EGF antibodies were determined using a validated ELISA. The confirmation of specificity used a floating cut point.

### 4.5. Safety/Tolerability Evaluations

Safety/tolerability evaluations including adverse events (AEs), physical examinations, clinical laboratory tests, vital sign measurements, and ECG were conducted In addition, ophthalmic examinations including best-corrected visual acuity, intraocular pressure, refractive error, slit-lamp examination, fundus examination, tear break-up time test, Schirmer’s test, and ocular surface disease index (OSDI) were conducted.

## 5. Conclusions

In conclusion, the dose range from 10–100 μg/mL solution of rhEGF eyedrops in single and multiple administrations was safe and well-tolerated in healthy male subjects. All ADRs were mild and transient. rhEGF eyedrops increased tear EGF level without effect on systemic exposure. Our findings justify further evaluation of the efficacy and safety of rhEGF eyedrops for corneal injury patients.

## Figures and Tables

**Table 1:** Summary of adverse drug reactions for rhEGF eyedrop and after single and multiple administration of rhEGF eyedrop and placebo.

	Dose Group				
	10 μg/mL (*n* = 6)	50 μg/mL (*n* = 6)	100 μg/mL (*n* = 6)	Placebo (*n* = 6)	Total (*n* = 24)
SAD study					
Total	3 (50) [3]	1 (16.7) [2]	1 (16.7) [1]	2 (33.3) [2]	7 (29.2) [8]
Eye disorders	3 (50) [3]	1 (16.7) [2]	1 (16.7) [1]	2 (33.3) [2]	7 (29.2) [8]
Corneal erosion	3 (50) [3]	1 (16.7) [1]		2 (33.3) [2]	6 (25) [6]
Dry eye		1 (16.7) [1]			1 (4.2) [1]
Eye pain			1 (16.7) [1]		1 (4.2) [1]
MAD study					
Total	1 (16.7) [1]	2 (33.3) [3]	2 (33.3) [2]	4 (66.7) [5]	9 (37.5) [11]
Eye disorders	1 (16.7) [1]	1 (16.7) [1]	2 (33.3) [2]	4 (66.7) [5]	8 (33.3) [9]
Corneal erosion	1 (16.7) [1]	1 (16.7) [1]	2 (33.3) [2]	3 (50) [4]	7 (29.2) [8]
Punctate keratitis				1 (16.7) [1]	1 (4.2) [1]
Gastrointestinal disorders		1 (16.7) [1]			1 (4.2) [1]
Stomatitis		1 (16.7) [1]			1 (4.2) [1]
		1 (16.7) [1]			1 (4.2) [1]
Headache		1 (16.7) [1]			1 (4.2) [1]

Notes: All data are presented as number of subjects (%) (number of cases). Abbreviations: SAD study, single ascending dose study; MAD study, multiple ascending dose study.

**Table 2:** Pharmacokinetic parameters of EGF in serum after single and multiple administration of rhEGF eyedrop.

	Dose Group			
	10 μg/mL (*n* = 6)	50 μg/mL (*n* = 6)	100 μg/mL (*n* = 6)	Placebo (*n* = 6)
SAD study				
C_max_ (μg/L)				
day −1	0.37 ± 0.08	0.37 ± 0.12	0.35 ± 0.07	0.39 ± 0.08
day 1	0.38 ± 0.10	0.36 ± 0.14	0.35 ± 0.07	0.34 ± 0.06
C_12h_ (μg/L)				
day −1	0.28 ± 0.12	0.26 ± 0.09	0.21 ± 0.06	0.23 ± 0.09
day 1	0.30 ± 0.12	0.32 ± 0.16	0.28 ± 0.06	0.30 ± 0.07
AUC_0-12h_ (μg∙h/L)				
day −1	3.94 ± 1.05	3.21 ± 1.89	2.42 ± 0.57	3.60 ± 0.73
day 1	3.06 ± 1.04	3.56 ± 1.34	3.03 ± 0.62	3.49 ± 0.56
T_max_ (h)				
day −1	3 (2–11.9)	7 (0.5–12)	2 (0–12)	2 (0.5–11.95)
day 1	0.25 (0–1)	0.38 (0.25–3.97)	0.48 (0–0.5)	3.98 (0–11.82)
MAD study				
C_max_ (μg/L)				
day −1	0.39 ± 0.14	0.33 ± 0.09	0.28 ± 0.08	0.32 ± 0.07
day 1	0.32 ± 0.11	0.36 ± 0.10	0.28 ± 0.08	0.38 ± 0.12
day 14	0.33 ± 0.11	0.33 ± 0.10	0.29 ± 0.06	0.37 ± 0.09
C_trough_ (μg/L)				
day −1	0.25 ± 0.11	0.22 ± 0.09	0.16 ± 0.04	0.24 ± 0.08
day 1	0.29 ± 0.09	0.27 ± 0.08	0.2 ± 0.05	0.29 ± 0.08
day 14	0.19 ± 0.09	0.25 ± 0.08	0.22 ± 0.1	0.25 ± 0.13
AUC_0-12h_ (μg∙h/L)				
day −1	3.76 ± 1.34	3.24 ± 0.72	2.50 ± 0.68	3.23 ± 0.92
day 1	3.07 ± 1.29	3.70 ± 1.05	2.59 ± 0.88	3.80 ± 1.18
day 14	3.13 ± 1.27	3.17 ± 0.87	2.94 ± 0.60	3.45 ± 1.25
T_max_ (h)				
day −1	11.68 (0.47–11.7)	11.72 (0.5–11.93)	7.93 (1–12)	2 (0.5–12)
day 1	1 (0–4)	4 (0.25–11.78)	6.23 (0–11.95)	3.01 (0.5–12)
day 14	2.01 (2–11.75)	6.9 (0–11.92)	0.5 (0–4)	7.87 (0–11.9)

Notes: Data presented as mean ± standard deviation. T_max_ was presented as median (min–max). Abbreviations: SAD study, single ascending dose study; MAD study, multiple ascending dose study; C_max_, the maximum concentration; C_max_, the minimum concentration; C_12h_, the observed serum concentration after 12 h of the administration; AUC_0-12h_, area under the serum concentration-time curve within time span 0 h to 12 h; T_max_, a time to reach maximum concentration.

**Table 3:** Pharmacokinetic parameters of EGF in serum after single and multiple administration of rhEGF eyedrop.

	Dose Group			
	10 μg/mL (*n* = 6)	50 μg/mL (*n* = 6)	100 μg/mL (*n* = 6)	Placebo (*n* = 6)
SAD study				
C_max_ (μg/L)				
day −1	2.45 ^1^	3.42 ^1^	6.09 ^1^	NA
day 1	3.24 ± 0.97 ^2^	1004.59 ± 1450.07 ^3^	678.37 ± 138.92 ^2^	4.11 ± 2.71 ^2^
C_12h_ (μg/L)				
day −1	2.08 ± 0.48 ^2^	1.43 ^1^	5.36 ± 1.00 ^2^	8.28 ^1^
day 1	1.07 ± 0.80 ^4^	2.14 ± 0.67 ^4^	4.61 ± 2.53 ^3^	3.25 ± 2.00 ^3^
AUC_0-12h_ (μg∙h/L)				
day −1	7.35 ^1^	20.33 ^1^	44.42 ^1^	NA
day 1	8.89 ± 4.42 ^2^	187.89 ± 233.79 ^3^	225.66 ± 45.55 ^2^	32.34 ± 15.88 ^2^
T_max_ (h)				
day −1	0.43 ^1^	3.92 ^1^	11.68 ^1^	NA
day 1	0.18 (0.17–0.18) ^2^	0.18 (0.17–0.2) ^3^	0.2 (0.18–0.22) ^2^	7 (2–12) ^2^
MAD study				
C_max_ (μg/L)				
day −1	5.05 ± 4.60 ^2^	NA	1.94 ^1^	NA
day 1	154.91 ^1^	112.00 ^1^	53.70 ± 53.09 ^2^	2.43 ± 0.24 ^2^
day 14	193.66 ± 88.04 ^2^	156.21 ^1^	803.53 ^1^	6.21 ^1^
C_trough_ (μg/L)				
day −1	7.75 ± 7.03	NA	3.60 ± 2.38	1.30 ± 0.36
day 1	2.18 ± 0.59	1.82 ± 0.31	3.87 ± 2.75	3.32 ± 1.56
day 14	1.30 ± 0.36	3.32 ± 1.56	8.29 ± 9.10	8.29 ± 9.10
AUC_0-12h_ (μg∙h/L)				
day −1	27.98 ± 17.56 ^2^	NA	16.22 ^1^	NA
day 1	70.65 ^1^	69.86 ^1^	30.91 ± 15.86 ^2^	14.82 ± 2.62 ^2^
day 14	85.2 ± 45.61 ^2^	49.19 ^1^	148.35 ^1^	42.35 ^1^
T_max_ (h)				
day −1	2.96 [2–3.92] ^2^	NA	0 ^1^	NA
day 1	0.18 ^1^	0.27 ^1^	0.19 [0.18–0.2] ^2^	0 [0–0] ^2^
day 14	0.22 [0.22–0.22] ^2^	0.23 ^1^	0.18 ^1^	4 ^1^

Notes: Data presented as mean ± standard deviation. T_max_ was presented as median (min–max). ^1^ n = 1, ^2^ n = 2, ^3^ n = 3, ^4^ n = 4. Abbreviations: SAD study, Single ascending dose study; MAD study, multiple ascending dose study; C_max_, the maximum concentration; C_max_, the minimum concentration; C_12h_, the observed serum concentration after 12 h of the administration; AUC_0-12h_, area under the serum concentration-time curve within time span 0 h to 12 h; T_max_, a time to reach maximum concentration.

## Data Availability

The datasets for this study will not be shared because it is possessed by Daewoong Bio Inc., Seoul, Korea.

## Supplementary Materials

The following supporting information can be downloaded at: https://www.mdpi.com/article/10.3390/ph15111312/s1, Table S1: Demographic characteristics; Table S2: Ocular surface health parameters after single and multiple administration of rhEGF eyedrop; Table S3: Incidence of anti-EGF antibodies after single and multiple administration of rhEGF eyedrop.
