# Supplementary material for: Safety, Tolerability, and Serum/Tear Pharmacokinetics of Human Recombinant Epidermal Growth Factor Eyedrops in Healthy Subjects
Source: Pharmaceuticals (Basel). 2022 Oct 24;15(11):1312. doi: 10.3390/ph15111312 (PMC9697941; doi:10.3390/ph15111312)
Supplement: Supplementary file 1 [file pharmaceuticals-15-01312-s001.zip › Table S1.pdf]

Table S1. Demographic characteristics

	Dose Group			
	**10 µg/mL (n=6)**	**50 µg/mL (n=6)**	**100 µg/mL (n=6)**	**Placebo (n=6)**
**SAD study**				
Age (years)	24.17 ± 1.17	26.14 ± 6.09	27.5 ± 7.2	31.67 ± 8.64
Body weight (kg)	75.05 ± 11.54	69.7 ± 7.46	64.9 ± 7.85	72.6 ± 5.51
Height (cm)	178.55 ± 9.09	170.64 ± 8.08	176.42 ± 3.47	173.53 ± 3.73
BMI (kg/m^2)	23.43 ± 1.95	23.96 ± 2.43	20.83 ± 2.09	24.13 ± 2.13
**MAD study**				
Age (years)	26.50 ± 5.54	32.17 ± 6.43	29.83 ± 7.86	29.00 ± 6.90
Body weight (kg)	71.95 ± 12.29	69.55 ± 7.34	74.68 ± 9.19	68.69 ± 6.17
Height (cm)	173.60 ± 3.70	172.33 ± 5.90	176.12 ± 3.69	174.00 ± 7.89
BMI (kg/m^2)	23.80 ± 3.40	23.38 ± 1.86	24.08 ± 2.91	22.73 ± 2.31

Data presented as mean ± standard deviation.

Abbreviations: SAD study, Single ascending dose study; MAD study, multiple ascending dose study; BMI, body mass index.
