# Supplementary material for: Safety, Tolerability, and Serum/Tear Pharmacokinetics of Human Recombinant Epidermal Growth Factor Eyedrops in Healthy Subjects
Source: Pharmaceuticals (Basel). 2022 Oct 24;15(11):1312. doi: 10.3390/ph15111312 (PMC9697941; doi:10.3390/ph15111312)
Supplement: Supplementary file 1 [file pharmaceuticals-15-01312-s001.zip › Table S2.pdf]

Table S2. Ocular surface health parameters after single and multiple administration of rhEGF eyedrop

	Dose Group			
	**10 µg/mL (n=6)**	**50 µg/mL (n=6)**	**100 µg/mL (n=6)**	**Placebo (n=6)**
**SAD study**				
Ocular surface disease index				
Screening	1.14 ± 2.78	3.83 ± 4.34	1.04 ± 1.74	7.99 ± 11.37
Post-study visit	0.42 ± 1.02	3.13 ± 4.32	1.83 ± 2.15	4.17 ± 10.21
Tear film break-up time (seconds)				
Screening	7.83 ± 0.41	7.14 ± 0.90	6.83 ± 0.75	6.92 ± 0.66
Post-study visit	7.00 ± 1.79	6.00 ± 2.10	7.50 ± 0.55	6.67 ± 1.21
Schirmer tests (mm)				
Screening	19.33 ± 8.24	24.14 ± 9.41	13.50 ± 8.17	16.33 ± 10.17
Post-study visit	24.33 ± 6.56	25.67 ± 9.44	14.67 ± 5.75	14.17 ± 6.35
**MAD study**				
Ocular surface disease index				
Screening	0.42 ± 1.02	1.39 ± 2.52	2.59 ± 3.26	3.4 ± 2.86
Post-study visit	3.58 ± 6.44	0.46 ± 1.13	2.62 ± 2.42	3.46 ± 5.23
Tear film break-up time (seconds)				
Screening	8.00 ± 0.63	8.00 ± 2.00	7.67 ± 0.52	6.79 ± 1.07
day 8	7.50 ± 0.84	7.83 ± 0.75	7.33 ± 0.52	7.25 ± 0.61
day 15	7.00 ± 1.26	7.67 ± 1.03	8.67 ± 1.97	6.58 ± 0.92
Post-study visit	6.33 ± 1.03	7.00 ± 0.63	8.17 ± 1.94	6.33 ± 1.83
Schirmer tests (mm)				
Screening	14.67 ± 4.59	14.17 ± 10.80	27.17 ± 10.52	20.64 ± 9.72
day 8	16.00 ± 10.18	18.67 ± 10.78	24.00 ± 13.19	16.58 ± 7.39
day 15	17.67 ± 9.85	16.83 ± 11.41	25.00 ± 12.49	17.92 ± 10.50
Post-study visit	17.17 ± 10.34	13.67 ± 8.82	24.17 ± 13.29	15.58 ± 10.44

Data presented as mean ± standard deviation.

Abbreviations: SAD study, Single ascending dose study; MAD study, multiple ascending dose study.
