# Supplementary material for: Safety, Tolerability, and Serum/Tear Pharmacokinetics of Human Recombinant Epidermal Growth Factor Eyedrops in Healthy Subjects
Source: Pharmaceuticals (Basel). 2022 Oct 24;15(11):1312. doi: 10.3390/ph15111312 (PMC9697941; doi:10.3390/ph15111312)
Supplement: Supplementary file 1 [file pharmaceuticals-15-01312-s001.zip › Table S3.pdf]

Table S3. Incidence of anti-EGF antibodies after single and multiple administration of rhEGF eyedrop

	Dose Group			
	**10 µg/mL (n=6)**	**50 µg/mL (n=6)**	**100 µg/mL (n=6)**	**Placebo (n=6)**
**SAD study**				
day 1	0/6(0)	0/6(0)	0/6(0)	1/5(16.7)
day 29-35	0/6(0)	0/6(0)	0/6(0)	0/6(0)
**MAD study**				
day 1	0/6(0)	0/6(0)	0/6(0)	0/6(0)
day 8	0/6(0)	0/6(0)	0/6(0)	0/6(0)
day 15	0/6(0)	0/6(0)	0/6(0)	0/6(0)
day 42-48	0/6(0)	0/6(0)	0/6(0)	0/6(0)

Data presented as the number of subjects with positive/negative (%) anti-EGF antibodies

Abbreviations: SAD study, Single ascending dose study; MAD study, multiple ascending dose study
